# Association between Bone Mineral Density and Severity of Chronic Kidney Disease

**DOI:** 10.1155/2020/8852690

**Published:** 2020-10-26

**Authors:** Jin-Feng Huang, Xuan-Qi Zheng, Xiao-Lei Sun, Xiao Zhou, Jian Liu, Yan Michael Li, Xiang-Yang Wang, Xiao-Lei Zhang, Ai-Min Wu

**Affiliations:** ^1^Department of Orthopaedics, The Second Affiliated Hospital and Yuying Children's Hospital of Wenzhou Medical University, The Second School of Medicine, Wenzhou Medical University, Wenzhou, Zhejiang 325027, China; ^2^Department of Orthopaedics, Tianjin Hospital, Tianjin, China; ^3^Ruian Institute of Quality and Technical Supervision and Inspection, Wenzhou 325000, China; ^4^Department of Nephrology, Rui Jin Hospital, Shanghai Jiao Tong University, School of Medicine, Shanghai 200025, China; ^5^Department of Neurosurgery and Oncology, University of Rochester Medical Center, School of Medicine and Dentistry, Rochester, NY 14642, USA

## Abstract

**Objective:**

We sought to evaluate the association between femoral neck (FN) and lumbar spine (LS) bone mineral densities (BMDs) with severity of chronic kidney disease (CKD) and prevalence of osteopenia or osteoporosis (OP) among the CKD group.

**Methods:**

Cross-sectional data from 11050 participants aged ≥20 years from the National Health and Nutrition Examination Survey (NHANES) were analyzed. Specifically, Pearson correlation was applied to analyze the relationship between BMD and estimated glomerular filtration rate (eGFR). General linear models (GLMs) were adjusted for potential confounders and used to analyze mean BMD, based on CKD and CKD stages.

**Results:**

FN BMD was positively correlated with the eGFR in the total and male CKD, but not in the female CKD population. LS BMD was not significantly associated with eGFR. After controlling for partial correlations, FN T-score was positively correlated with the eGFR in the total at-risk population. According to FN BMD, OP prevalence was positively associated with CKD stage. However, according to LS BMD, there was no significant association between OP and CKD stage.

**Conclusion:**

Our results may explain the higher prevalence of hip fracture, relative to that of the spine, among CKD patients and generate meaningful insights to guide care, prevention, and treatment regimens for CKD patients. However, the fact that this was a cross-sectional study may limit the possibility of drawing concrete conclusions. Nevertheless, these findings open up a new frontier for further studies to uncover the higher decrease of FN BMD compared to LS BMD among CKD cases.

## 1. Introduction

Chronic kidney disease (CKD) is a common global public health problem [1–3]. The disease is defined when a person exhibits kidney damage or glomerular filtration rate (GFR) of <60 ml/min/1.73 m^2^ for 3 months or more [4]. Based on GFR and albuminuria, the National Kidney Foundation's Kidney Disease Outcomes Quality Initiative (KDOQI) has recommended classification of CKD into different stages [5], since patients at different stages of the disease exhibit distinct prognosis as well as body states.

Bone fractures are a common occurrence in patients with CKD [6, 7]. For example, osteoporosis (OP), a systemic skeletal disease manifested as low bone mass and destruction of the bone tissue microenvironment, has been described [8]. In fact, OP patients always exhibit an increase in bone fragility and are more prone to fractures [9]. Although CKD and OP are two different diseases, they both negatively affect bone metabolism [10–12]. For instance, mineral and bone disorders were a common occurrence in CKD patients [13], leading to coining of the term of CKD-mineral and bone disorder (CKD-MBD) [14]. Generally, CKD-MBD means a systemic disorder containing one or combination of the following: (1) abnormalities in calcium, phosphorus, parathyroid hormone (PTH), and vitamin D metabolism; (2) calcification of vascular or other soft tissues; and/or (3) abnormalities in bone turnover, mineralization, or strength. These phenomena lead to bone disease and increased risk of fractures [14, 15].

Previous studies have shown that CKD occurrence can increase the risk of bone disease and fractures [16–18]. For example, CKD stages 3a–5D were found to have low bone mineral density (BMD) and 1.5 to 2-fold higher risk of fractures than in the general population [19]. However, conflicting results have been reported with regard to the complex association between BMD and kidney function [20–23], necessitating further studies.

The dual-energy X-ray absorptiometry (DXA) is a good predictor of fracture risk and the gold standard method for measuring BMD. In 2009, Kidney Disease: Improving Global Outcomes (KDIGO) guidelines were recommended for routine BMD testing [24]. However, these guidelines were updated in 2017 allowing their use in predicting the risk of fracture in CKD patients [25]. This change of guidelines indicates that using BMD and T-score screening for osteopenia and OP can be used for CKD patients.

Prevalence of osteopenia and OP, as well as the association between BMD and renal function among American CKD patients, is not well understood [26]. In the current study, we thoroughly analyze the association, adjusted for multiple potential confounders, between BMD and eGFR among American CKD patients. We used many statistical methods such as general liner model, Pearson correlation, and partial correlations to assess the relationship. Moreover, we also included multiple potential confounders, especially medication taken history, into analysis. Besides, the large number of patients (>10, 000) studied constitute one of the main strengths of our analysis. The statistical power of our study was therefore relatively high.

## 2. Methods

### 2.1. Data Sources and Study Participants

In this study, we collected data from the National Health and Nutrition Examination Survey (NHANES) database. The NHANES, conducted by the National Center for Health Statistics (NCHS), aims to assess the health and nutritional status of the US population [27]. Prior to data collection, all NHANES survey protocols are required to be evaluated by the NCHS Research Ethics Board, and informed consent was signed [28]. The present study extracted data from four NHANES cycles, including the periods between 2005–2010 and 2013-2014. In these cycles, BMDs of the femoral neck and lumbar spine were measured by DXA. For this study, no ethical approval was required, since we analyzed public data.

The analytic cohorts were derived from adults, aged 20 years and older, in NHANES. Respondents who lacked valid FN BMD and LS BMD data or did not have data for at least two lumbar vertebrae were excluded from the analysis [29].

### 2.2. Retrieval of Clinical, Laboratory, and BMD Data

We retrieved demographic and comorbidity data, including age, sex, BMI, diabetes, coronary heart disease, arthritis, congestive heart failure, stroke, chronic bronchitis, and smoking history, as well as laboratory findings (e.g., albumin, alkaline phosphatase (ALP), calcium, creatinine, phosphorus, vitamin D, and glycohemoglobin levels) according to previous studies [30, 31]. Other variables collected included fracture history (hip, wrist, spine, and other sites), menopause status, status, and types of medication taken such as antiresorptive drugs (e.g., alendronate, risedronate, ibandronate, raloxifene, and calcitonin), angiotensin-converting enzyme inhibitors (ACEI), estrogen, loop diuretics, and glucocorticoids, as well as research year cycles and DXA results.

### 2.3. Measurement and Interpretation of Bone Mineral Densities

In NHANES 2005–2010, femoral neck and posterior-anterior lumbar spine scans were examined using Hologic QDR 4500A fan-beam, while those from 2013 to 2014 were measured by Hologic Discovery® A (Hologic Inc., Marlborough, MA) densitometers [32]. In the 2005–2010 segment, Hologic Discovery v12.4 was used for analyzing femur scans, whereas the spine scans were assessed using APEX v3.0. On the other hand, both femur and spine scans in the 2013-2014 segment were analyzed using APEX v4.0. Previous studies have revealed no significant differences in mean BMDs analyzed using Hologic Discovery v12.4 and APEX v4.0 [33].

Studies have also indicated that male subjects, subjects older than 50 years, and postmenopausal females exhibit marked bone mineral loss; therefore, this population is more prone to osteoporotic fracture [34, 35]. Based on this, we defined male participants aged ≥50 and postmenopausal women as at-risk population. For this population, we calculated T-scores before analyzing the association between OP prevalence and CKD stages. T-scores were defined as BMD respondent minus the mean BMD of the reference group and then divided by standard deviation (SD) of the reference group. We considered 30-year-old white females from the DXA manufacturer reference database, and 20–29-year-old non-Hispanic white females, from NHANES III, as the reference groups for lumbar spine and femoral neck, respectively [36]. In addition, we defined osteopenia as a T-score between −1.0 and −2.5 and OP as a T-score ≤ −2.5 according to the criteria recommended by the World Health Organization (WHO) [37, 38]. We also defined “low T-score” as having the lowest T-score ≤ −1, that is, within the range of osteopenia and OP. So, using low T-score can find low bone mass patients more sensitive. OP prevalence and low bone mass were calculated separately, for femur neck and lumbar spine.

### 2.4. Measurement of Chronic Kidney Disease

To calculate estimated glomerular filtration rate (eGFR), we adopted the Chronic Kidney Disease Epidemiology Collaboration equation [39]. Briefly, albuminuria was defined as the ratio of urinary albumin to creatinine (ACR) [40]. CKD was defined as ACR ≥30 mg/g or eGFR blow 60 mL/min/1.73 m^2^ [41]. We divided CKD participants into different stages, according to the guidelines provided by the KDIGO for further study [5]. Stage 1 CKD was defined as cases with eGFR ≥90 mL/min/1.73 m^2^ and ACR ≥30 mg/g, stage 2 CKD as eGFR 60 to 89 mL/min/1.73 m^2^ and ACR ≥30 mg/g, stage 3 CKD as eGFR 30 to 59 mL/min/1.73 m^2^, and stage 4 as those with eGFR <30 mL/min/1.73 m^2^.

### 2.5. Anthropometric Measurement

Weight (kg) and height (cm) of the respondents were measured while they were dressed in light clothing without shoes and their BMI defined as the ratio of weight to the square of the height (kg/m^2^). Thereafter, their BMIs were divided into three groups (<25, 25 to <30, ≥30), whereas smoking history was classified into two groups (<100 cigarettes and ≥100 cigarettes).

### 2.6. Statistical Analysis

Four NHANES cycles (2005-2006, 2007-2008, 2009-2010, and 2013-2014) were pooled before analysis. Participant characteristics were first compared between CKD and the control group. All continuous variables evaluated their distribution firstly. For normal variable distribution variables, we conducted Student's *t*-test, whereas nonnormal variable distribution ones were subjected to the nonparametric tests. The categorical variables were compared using the Chi-square test. Characteristics of participants among subgroups at different CKD stages were compared using analysis of variance (ANOVA), for continuous variables, and the Chi-square test for categorical ones.

A Pearson correlation matrix was used to analyze the relationship between BMD and eGFR. Another Pearson correlation analysis was also done after classifying different groups according to sex to investigate specific associations. We then performed partial correlations, while controlling for race, age, and sex (partial correlation 1) and race, age, sex, and BMI (partial correlation 2) and for all covariates (age, sex, BMI, ALP, albumin, total calcium, phosphorus, 25(OH)D3, glycohemoglobin, diabetes, coronary heart disease, arthritis, congestive heart failure, stroke and chronic bronchitis, fracture history, menopause status, medication, and research year cycles) (partial correlation 3). During gender-based subgroup analysis, we excluded sex variate from controlling analysis.

The relationships between BMD and CKD stage were further analyzed in patients, using generalized linear models (GLM), with adjustment for multiple covariates. Age, sex, and BMI were considered essential covariates, owing to their marked association with CKD and OP [42–45]. Model 1 was adjusted by age, sex, and race and Model 2 by age, sex, race, and BMI, whereas Model 3 was adjusted for age, sex, BMI, ALP, albumin, total calcium, phosphorus, 25(OH)D3, glycohemoglobin, diabetes, coronary heart disease, arthritis, congestive heart failure, stroke, and chronic bronchitis, as well as fracture history, menopause status, medication, and research year cycles. During sex-based subgroup analyses, we excluded gender from model controlling. The multicollinearities between covariates were evaluated by correlation analyses and collinearity statistics. *Post hoc* analyses were conducted using GLM or logistic regression setting, with stage 1 CKD taken as the reference subgroup.

All statistical analyses were performed in SPSS software (version 18; IBM Corp., USA). Since multiple testing may result in type I error, we considered *P* < 0.01 as statistically significant during investigations of several endpoints or performed several statistical tests on the same data, such as ANOVA and *post hoc* analyses [46]. We considered *P* < 0.05 to be statistically significant across results from Student's *t*-tests and Chi-square, GLM, and logistic regression model.

## 3. Results

### 3.1. Relationship between BMD and the Presence of CKD

A total of 11,050 respondents, 1,572 with and 9,478 without CKD were recruited in our study between 2005–2010 and 2013-2014. A summary of their characteristics is outlined in Table 1. The mean FN BMD was lower in the CKD group compared to that without (*P* < 0.001), whereas no significant difference was observed between the groups with regard to LS BMD (*P*=0.114).

OP prevalence for FN BMD was significantly higher in the CKD group (7.2%) compared to the group without (3.0%). In addition, prevalence of OP calculating for FN BMD was more common in female CKD patients (12.3%). Conversely, OP prevalence for LS BMD revealed no significant differences between the two groups. A detailed description for prevalence of osteopenia, OP, and low T-scores across the 2 groups is outlined in Table 2.

### 3.2. Association between BMD and eGFR

**Figure 1:**
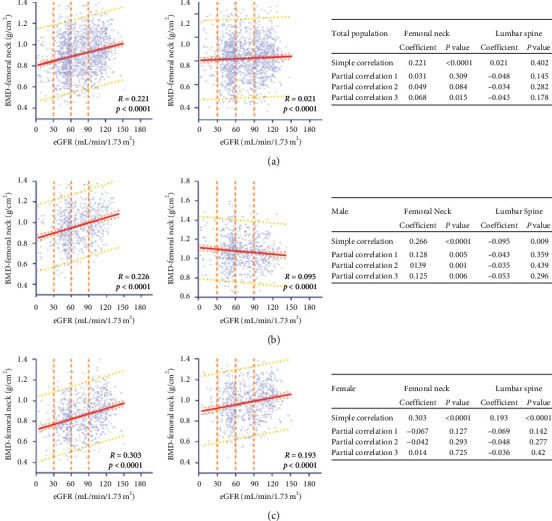
Scatterplot, Pearson correlation, and partial correlations between BMD and eGFR in total population, males, and females (a, b, and c, respectively). BMD is presented by a logarithmic scale along the *y*-axis and eGFR along the *x*-axis. Regression lines are shown with 95% CIs (orange lines) and 95% prediction interval (yellow lines). BMD, bone mineral density; CKD: chronic kidney disease; eGFR: estimated glomerular filtration rate.

We further investigated the relationship between low BMD and low eGFR. Scatterplots and Pearson correlation coefficients revealed a positive correlation between FN BMD and eGFR in the CKD population (*r* = 0.221; *P* < 0.0001 in the total population; *r* = 0.303; *P* < 0.0001 in the female population; *r* = 0.266; *P* < 0.0001 in the male population; Figures 1(a)–1(c)). However, there is no significant relationship between LS BMD and eGFR (*r* = 0.021; *P*=0.402 in the total population; Figures 1(d) and 1(f)). After performing partial correlations to control for race, age, and sex (partial correlation 1); race, age, sex, and BMI (partial correlation 2); and all covariates (partial correlation 3), FN BMD was positively correlated with the eGFR in the total and male CKD populations, but not in female CKD population (Figure 1(g)).

### 3.3. Association between BMD and CKD Severity

Mean FN and LS BMDs according to subgroup by CKD stage are presented in Table 3. Summarily, unadjusted and adjusted FN BMDs were significantly different between subgroups based on the CKD stage. However, no significant differences were obtained for LS BMD (Table 3). *Post hoc* analyses showed that unadjusted FN BMD in CKD stages 2, 3, and 4 were significantly different with CKD stage 1. After adjusting for race, age, and sex, FN BMD still showed a negative relationship with CKD stage, although LS BMD had a positive relationship with CKD stage (Model 1 for FN and LS BMDs in Table 3). Further adjusting for BMI and other covariates revealed similar results, with a negative relationship between FN BMD and CKD stage as well as a positive correlation between LS BMD and CKD stage (Models 2 and 3 for FN and LS BMDs in Table 3). Moreover, the relationship between LS BMD and CKD stage remained insignificant even after adjusting for covariates (Models 1, 2, and 3 for LS BMD in Table 3).

### 3.4. Association between T-Score and eGFR in the At-Risk Population

**Figure 2:**
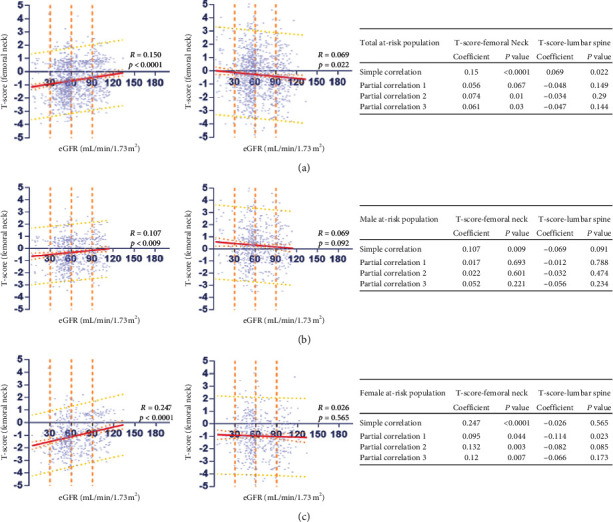
Scatterplot, Pearson correlation, and partial correlations between T-score and eGFR in the total at-risk population, male at-risk population, and female at-risk population (a, b, and c, respectively). T-score is presented by a logarithmic scale along the *y*-axis and eGFR along the *x*-axis. Regression lines are shown with 95% CIs (orange lines) and 95% prediction interval (yellow lines). BMD, bone mineral density; eGFR: estimated glomerular filtration rate; the at-risk population, male participants aged ≥50 years, and postmenopausal female.

We further investigated whether low T-scores were associated with low eGFR in the at-risk population. Scatter plots and Pearson correlation coefficients revealed a positive correlation between FN T-score and eGFR in the at-risk population (Figures 2(a)–2(c)). However, there is no significant relationship between LS T-score and eGFR in female or male subgroup (*r* = −0.026; *P*=0.565 in the female at-risk population; *r* = −0.069; *P*=0.091 in the male at-risk population; Figures 2(e) and 2(f)) After controlling for partial correlations, we observed a positive correlation between FN T-score and the eGFR in the total at-risk population and a negative relationship between LS T-score and eGFR (Figure 2(g)).

### 3.5. Association between OP and Stage in the At-Risk CKD Population

Thereafter we investigated the relationship between OP prevalence and CKD stage in the at-risk population. Results revealed a positive correlation between OP prevalence according to FN BMD and CKD stage, whereas that according to LS BMD was not significant (Table 4). OP prevalence based on FN BMD in female or male subgroups still showed a significant association with CKD, whereas according to LS BMD it showed no significant association with CKD stage in either female or male subgroups.

## 4. Discussion

Our findings revealed that adjusted FN BMDs have a significant positive association with eGFR and the stage of CKD. Besides, the association is linear, depending on the stage of CKD. However, LS BMD is neither significantly associated with eGFR nor the stage of CKD. In addition, prevalence of OP in FN, but not LS BMD, showed a positive association with the stage of CKD.

Our results further indicated that CKD prevalence causes a significantly lower FN BMD, but not LS BMD. Unadjusted FN BMD was positively related to eGFR and stage of CKD, and the relationship was still statistically significant after adjusting for multiple other confounders. However, the relationship between LS BMD and eGFR or stage of CKD was not significant after adjusting. These findings overrule accuracy of assuming a simple positive or negative relationship between eGFR and BMD, owing to an effect of potential factors such as aging process, lifestyle, medical conditions, and different sites of bone. However, these findings were partially consistent with a previous study [30]. Besides, after grouping by sex and adjusting for other confounders, we found that the association was still significant in males, but not females. A similar result was reported by Ishani et al. who found a significant association between eGFR and BMD in males [47]. This inconsistency may be due to other confounders that potentially exert greater impacts on females than males.

The association between unadjusted FN BMD and the stage of CKD was significantly negatively linear, whereas that of FN BMD (for participants with stages 2, 3, and 4) was significantly different in participants with stage 1. However, after adjusting for other confounders, only the FN BMD in participants with stages 3 and 4 was significantly different from those at stage 1. This result is consistent with previous studies in which BMD was found to decrease in stages 3–5 [21, 48, 49]. With regard to CKD progression, several hormonal and biochemical changes such as hyperphosphatemia, hypocalcemia, increased fibroblast growth factor 23 (FGF-23), and PTH levels as well as vitamin D concentration abnormalities have been suggested [50]. Occurrence of these changes *in vivo* may explain the above result. Previous studies have demonstrated elevated levels of circulating osteoprotegerin (OPG) and receptor activator of nuclear factor-ĸB ligand (RANKL) in OP patients [51–54]. OPG was a factor associated with aortic stiffness and cardiovascular mortality in CKD patients [55]. Additionally, excessive bone resorption contributed to hyperphosphatemia with stimulation of heterotopic mineralization such as vascular calcification [56]. In our study, we hypothesize that CKD progression led to loss of bone mass which subsequently may increase the risk of aortic calcification and cardiovascular mortality in CKD patients. It is a vicious circle. This disorder of systems biology links skeletal and kidney dysfunction to the risk of cardiovascular diseases and mortality through the CKD-MBD [57]. Future studies should therefore focus on the kidney-skeletal-cardiovascular axis which potentially plays an important role in prognosis and survival of CKD-MBD patients. Results generated from this may lead to development of an important therapeutic target for improving long-term outcomes of CKD-MBD patients.

Chen et al. reported that prevalence of spinal fractures in CKD patients was similar to the general population [58]. Our results partly support this finding. Specifically, we found eGFR to be independently associated with FN BMD and CKD, but LS BMD was not significantly associated with eGFR. The factors that influenced the higher FN BMD during CKD progression but did not influence LS BMD remain unclear. This result may be explained by the fact that the hip has more cortical bone than, which is more impaired compared to, trabecular of the bone in CKD patients [59–61].

Previous studies have drawn different conclusion regarding Asian populations [20, 31, 62]. Results from the current study differ from the aforementioned reports, because ours were based on different ethnic population. Previous studies have showed that the effect of height, weight, body composition, environmental factors, and lifestyle may all result in ethnic differences in bone mass and eGFR [36, 63–67]. Thus, evaluating association between BMD and other factors based on different populations may reveal different results. Levey et al. demonstrated that the Chronic Kidney Disease Epidemiology Collaboration (CKD-EPI) equation overcomes some of the limitations of the Modification of Diet in Renal Disease (MDRD) equation and therefore has important implications for public health and clinical practice [68]. In the last decade, CKD-EPI equation was extensively used to calculate eGFR. However, recently, no studies have recently used this new equation to describe the association between eGFR and BMD in American CKD groups. It is, therefore, necessary to update our knowledge regarding the CKD-EPI equation. Generally, our study is more suitable for US populations and follows the recent advances in this domain.

The present study had some limitations. Firstly, we used a cross-sectional design in the study, which may have caused the direction of causality not to be formally assessed. Secondly, the CKD-EPI equation incorporated age, sex, and race in its calculation, which may all have impacted the BMD. It is, therefore, possible that entry of these variables may alter the association between CKD and other variables or create interaction terms in multivariate models. Furthermore, there may be a little imprecise by using eGFR, calculating by CKD-EPI equation, to represent GRF. However, to data, CKD-EPI equation remains the most accurate and widely used method for estimating GFR. Thirdly, we lacked data for hormones and markers involved in bone metabolism. Therefore, pathophysiology underlying the association between eGFR of CKD patients and BMD of different sites could not be elucidated, and, as a result, we could not evaluate the type of bone impairment. In future, prospective controlled trials should be conducted to reveal the underlying pathophysiological mechanisms and causal factors involved in different bone sites during CKD progression. Fourthly, our study was based on an American population, which does not represent the entire CKD population worldwide. Fifthly, we did not consider LS and FN status, in terms of presence of CKD and individual radiographic features, which might confound the BMD results. Finally, selection bias may be present due to dropped data and the nonresponse rate.

## 5. Conclusion

In summary, we assessed the association between BMD and eGFR in patients with CKD. We found that FN BMD was decreased in CKD patients. Further analysis showed that FN BMD was significantly positively associated with eGFR or stage of CKD, but there was no significant association between LS BMD and eGFR or stage of CKD. After adjusting for potential confounders, the association between FN BMD and severity of CKD persisted. These results may explain why the prevalence of hip fracture is much higher than that of spine fracture among patients with CKD. This is important as it offers a guide for effective care, prevention, and treatment of patients with CKD. However, because this was a cross-sectional study, the strength of our results is limited. Nevertheless, our results provide new ideas further to understand why FN BMD is much lower than LS BMD in patients with CKD.

## Figures and Tables

**Table 1:** Comparison of sample characteristics between those with chronic kidney disease according to current chronic kidney disease status.

	Healthy (*N* = 9478)	CKD group (*N* = 1572)	*P* value^1^	Stage I (*N* = 532)	Stage II (*N* = 381)	Stage III (*N* = 597)	Stage IV (*N* = 62)	*P* value^2^
*Age, years (mean* *±* *SD)*	45.71 ± 15.38	60.42 ± 16.68	<0.001	45.44 ± 13.68	63.38 ± 13.48	71.18 ± 10.39	67.11 ± 12.68	<0.0001

*eGFR (mL/min/1.73 m* ^*2*^)	98.21 ± 18.01	74.76 ± 28.79	<0.001	108.18 ± 11.88	76.03 ± 8.67	50.07 ± 7.63	17.90 ± 8.69	<0.0001

*BMI, kg/m* ^*2*^ *(mean* *±* *SD)*	27.99 ± 5.55	28.69 ± 5.94	<0.001	29.14 ± 6.85	28.64 ± 5.62	28.36 ± 5.25	28.22 ± 5.54	0.148

*FN BMD (g/cm* ^*2*^)	0.96 ± 0.17	0.91 ± 0.18	<0.0001	0.96 ± 0.17	0.90 ± 0.19	0.88 ± 0.17	0.82 ± 0.19	<0.0001

*LS BMD (g/cm* ^*2*^)	1.03 ± 0.15	1.03 ± 0.18	0.114	1.04 ± 0.15	1.02 ± 0.18	1.02 ± 0.19	1.05 ± 0.20	0.227

*Laboratorial test*								
Albumin (g/dl)	4.27 ± 0.31	4.16 ± 0.34	<0.001	4.19 ± 0.36	4.19 ± 0.33	4.14 ± 0.31	3.93 ± 0.49	<0.0001
Alkaline phosphatase (U/L)	68.11 ± 22.68	74.96 ± 31.41	<0.001	75.48 ± 38.03	75.83 ± 27.45	71.91 ± 25.13	94.47 ± 38.26	<0.0001
Total calcium (mg/dL)	9.45 ± 0.35	9.46 ± 0.41	0.424	9.44 ± 0.39	9.51 ± 0.40	9.46 ± 0.39	9.26 ± 0.55	<0.0001
Phosphorus (mg/dL)	3.76 ± 0.55	3.77 ± 0.62	0.724	3.71 ± 0.56	3.69 ± 0.57	3.77 ± 0.56	4.63 ± 1.13	<0.0001
25(OH)D3	26.02 ± 9.8	26.81 ± 9.7	0.123	26.72 ± 9.6	27.22 ± 9.5	26.89 ± 9.6	26.62 ± 10.1	0.248
Glycohemoglobin, %	5.56 ± 0.84	6.21 ± 1.60	<0.001	6.34 ± 1, 96	6.35 ± 1.73	6.01 ± 1.05	6.26 ± 1.69	0.01

*Gender (n, %)*			0.091					<0.0001
Male	4782 (50.5%)	757 (48.2%)		205 (38.5%)	197 (51.7%)	324 (54.3%)	31 (50.0%)	
Female	4696 (49.5%)	815 (51.8%)		327 (61.5%)	184 (48.3%)	273 (45.7%)	31 (50.0%)	

*Race (n, %)*			<0.001					<0.0001
Non-Hispanic white	1915 (20.2%)	274 (17.4%)		157 (29.5%)	62 (16.3%)	50 (8.4%)	5 (8.1%)	
Non-Hispanic black	903 (9.5%)	132 (8.4%)		58 (10.9%)	35 (9.2%)	37 (6.2%)	2 (3.2%)	
Mexican–American	4486 (47.3%)	768 (48.9%)		170 (32.0%)	187 (49.1%)	389 (65.2%)	22 (35.5%)	
Other Hispanic	1743 (18.4%)	343 (21.8%)		120 (22.6%)	83 (21.8%)	109 (18.3%)	31 (50.0%)	
Other race	431 (4.5%)	55 (3.5%)		27 (5.1%)	14 (3.6%)	12 (2.0%)	2 (3.2%)	

*Fracture history (n, %)*								
Previous hip fracture	100 (1.1%)	31 (2.0%)	0.008	8 (1.5%)	10 (2.6%)	13 (2.2%)	—	0.615
Previous wrist fracture	825 (8.7%)	156 (9.9%)	0.290	39 (7.3%)	44 (11.5%)	66 (11.1%)	7 (11.3%)	0.159
Previous spine fracture	150 (1.6%)	29 (1.8%)	0.510	8 (1.5%)	7 (1.8%)	14 (2.3%)	—	0.714
Previous other site fractures	1940 (20.5%)	368 (23.4%)	0.025	96 (18.0%)	97 (25.5%)	157 (26.3%)	18 (29.0%)	0.022

*Lifetime smoking (n, %)*			0.011					0.247
<100 cigarettes	4314 (45.5%)	770 (49.0%)		246 (46.2%)	187 (49.1%)	301 (50.4%)	36 (58.1%)	
≥100 cigarettes	5164 (54.5%)	802 (51.0%)		286 (53.8%)	194 (50.9%)	296 (49.6%)	26 (41.9%)	

*Menopause (n, %)*	1731 (18.3%)	506 (32.2%)	<0.001	98 (18.4%)	131 (34.4%)	254 (42.5%)	23 (37.1%)	<0.0001

*Medication (n, %)*								
Antiresorptive drugs	103 (1.1%)	43 (2.7%)	<0.001	3 (0.6%)	12 (3.1%)	27 (4.5%)	1 (1.6%)	<0.0001
ACEI	815 (8.6%)	391 (24.9%)	<0.001	78 (14.7%)	106 (27.8%)	188 (31.5%)	19 (30.6%)	<0.0001
Estrogen	91 (1.0%)	18 (1.1%)	0.892	1 (0.2%)	6 (1.6%)	10 (1.7%)	1 (1.6%)	<0.0001
Loop diuretics	95 (1.0%)	126 (8.0%)	<0.001	8 (1.5%)	24 (6.3%)	74 (12.4%)	21 (33.9%)	<0.0001
Glucocorticoids	381 (4.0%)	108 (6.9%)	<0.001	28 (5.3%)	18 (4.7%)	52 (8.7%)	10 (16.1%)	<0.0001

*Known comorbidities (n, %)*								
Diabetes mellitus	645 (6.8%)	399 (25.4%)	<0.001	125 (23.5%)	113 (29.7%)	132 (22.1%)	29 (46.8%)	<0.0001
Coronary heart disease	206 (2.2%)	141 (9.0%)	<0.001	13 (2.4%)	43 (11.3%)	69 (11.6%)	16 (25.8%)	<0.0001
Arthritis	1995 (21.0%)	570 (36.3%)	<0.001	112 (21.1%)	146 (38.3%)	281 (47.1%)	31 (50.0%)	<0.001
Congestive heart failure	89 (0.9%)	74 (4.7%)	<0.001	9 (1.7%)	24 (6.3%)	31 (5.2%)	10 (16.1%)	<0.0001
Stroke	176 (1.9%)	113 (7.2%)	<0.001	16 (3.0%)	28 (7.3%)	55 (9.2%)	14 (22.6%)	<0.0001
Chronic bronchitis	471 (5.0%)	129 (8.2%)	<0.001	32 (6.0%)	31 (8.1%)	59 (9.9%)	7 (11.3%)	0.061
*Year cycles (n, %)*								
2005–2006	2368 (25.0%)	447 (28.4%)	<0.001	126 (23.7%)	97 (25.5%)	206 (34.5%)	18 (29.0%)	<0.0001
2007–2008	2662 (28.1%)	492 (31.3%)	<0.001	180 (33.8%)	113 (29.7%)	183 (30.7%)	16 (25.8%)	0.384
2009–2010	3068 (32.4%)	389 (24.7%)	<0.001	143 (26.9%)	89 (23.4%)	143 (24.0%)	14 (22.6%)	0.562
2013–2014	1380 (14.6%)	244 (15.5%)	0.319	83 (15.6%)	82 (21.5%)	65 (10.9%)	14 (22.6%)	<0.0001

Values are the means ± SE or *n* (%), as appropriate. *P* values^1^ by Student's *t* tests or nonparametric tests for continuous variables and the Chi-square test for categorical variables; *P* < 0.05 was considered statistically significant. *P* values^2^ by analysis of variance (ANOVA) for continuous variables and the Chi-squared test for categorical variables; *P* < 0.01 was considered statistically significant. FN, femoral neck; LS, lumbar spine; BMD, bone mineral density; CKD: chronic kidney disease; NHANES: the National Health and Nutrition Examination Survey; eGFR: estimated glomerular filtration rate; 25(OH)D3, 25-hydroxyvitamin D3.

**Table 2:** Prevalence of osteopenia, osteoporosis, or low t-score in the at-risk population according to the presence of CKD (male participants aged ≥ 50 years and postmenopausal women).

	Healthy (*N* = 3577)	CKD group (*N* = 1104)	*P* value
Femoral neck			
Total population			
Osteopenia (*n*, %)	1076 (30.1%)	382 (34.6%)	<0.001
Osteoporosis (*n*, %)	108 (3.0%)	79 (7.2%)	0.005
Low T-score (*n*, %)	1184 (33.1%)	461 (41.8%)	<0.001
Female			
Osteopenia (*n*, %)	656 (37.9%)	214 (42.3%)	0.074
Osteoporosis (*n*, %)	91 (5.3%)	62 (12.3%)	<0.001
Low T-score (*n*, %)	747 (43.2%)	276 (54.5%)	<0.001
Male			
Osteopenia (*n*, %)	420 (22.8%)	168 (28.1%)	0.008
Osteoporosis (*n*, %)	17 (0.9%)	17 (2.8%)	<0.001
Low T-score (*n*, %)	437 (23.7%)	185 (30.9%)	<0.001

Lumbar spine			
Total population			
Osteopenia (*n*, %)	1064 (29.7%)	274 (24.8%)	0.063
Osteoporosis (*n*, %)	254 (7.1%)	97 (8.8%)	0.002
Low T-score (*n*, %)	1318 (36.8%)	371 (33.6%)	<0.05
Female			
Osteopenia (*n*, %)	661 (38.2%)	176 (34.8%)	0.164
Osteoporosis (*n*, %)	200 (11.6%)	81 (16.0%)	0.008
Low T-score (*n*, %)	861 (49.7%)	257 (50.8%)	0.678
Male			
Osteopenia (*n*, %)	403 (21.8%)	98 (16.4%)	0.004
Osteoporosis (*n*, %)	54 (2.9%)	16 (2.7%)	0.750
Low T-score (*n*, %)	457 (24.8%)	114 (19.1%)	0.004

*P* values by the Chi-square test; *P* < 0.05 was considered statistically significant. The at-risk population, male participants aged ≥50 years, and postmenopausal women; low T-score and T-score ≤ -1.

**Table 3:** Mean BMD (g/cm^2^) according to the severity of CKD.

	Stage I (*N* = 532)	Stage II (*N* = 381)	Stage III (*N* = 597)	Stage IV (*N* = 62)	*P* for trend
*Femoral neck BMD*					
Unadjusted	0.96 ± 0.01	0.90 ± 0.01^†^	0.88 ± 0.01^†^	0.82 ± 0.02^†^	<0.001
Model 1	0.94 ± 0.01	0.90 ± 0.01^*∗*^	0.90 ± 0.01^*∗*^	0.84 ± 0.02^†^	0.002
Model 2	0.93 ± 0.01	0.90 ± 0.01^*∗*^	0.90 ± 0.01^*∗*^	0.84 ± 0.02^†^	0.006
Model 3	0.94 ± 0.01	0.91 ± 0.01^*∗*^	0.90 ± 0.01^*∗*^	0.86 ± 0.03^†^	0.033

*Lumbar spine BMD*					
Unadjusted	1.04 ± 0.01	1.02 ± 0.01	1.02 ± 0.01	1.05 ± 0.02	0.227
Model 1	1.02 ± 0.01	1.02 ± 0.01	1.03 ± 0.01	1.05 ± 0.02	0.727
Model 2	1.03 ± 0.01	1.02 ± 0.01	1.03 ± 0.01	1.05 ± 0.02	0.714
Model 3	0.99 ± 0.01	0.98 ± 0.01	0.98 ± 0.01	0.98 ± 0.03	0.917

Values are the means ± SE. *P* for trend values by general linear models; *P* < 0.05 was considered statistically significant. ^*∗*^*P* < 0.01 and ^†^*P* < 0.001 by *post hoc* analyses, CKD stage 1 subgroup as reference, and *P* < 0.01 was considered statistically significant. Model 1, adjusted for age, sex, and race. Model 2, adjusted for age, sex, race, and BMI. Model 3, adjusted for age, sex, BMI, ALP, total calcium, albumin, phosphorus, 25(OH)D3, glycohemoglobin, diabetes, coronary heart disease, arthritis, congestive heart failure, stroke and chronic bronchitis, fracture history, menopause status, medication, and research year cycle. BMD, bone mineral density; CKD, chronic kidney disease; 25(OH)D3, 25-hydroxyvitamin D3; BMI, body mass index; ALP, alkaline phosphatase.

**Table 4:** Prevalence of osteoporosis in the at-risk population according to the severity of CKD.

	Stage I (*N* = 196)	Stage II (*N* = 295)	Stage III (*N* = 560)	Stage IV (*N* = 53)	*P* for trend
*Osteoporosis-femoral neck (n, %)*					
Total population	6 (3.1%)	19 (6.4%)	46 (8.2%)	9 (17.0%)^†^	0.002
Female	4 (4.1%)	16 (12.2%)	36 (14.2%)^†^	5 (21.7%)^†^	0.008
Male	2 (2.0%)	3 (1.8%)	10 (3.3%)	4 (13.3%)^†^	0.037

*Osteoporosis-lumbar spine (n, %)*					
Total population	14 (7.1%)	26(8.8%)	54 (9.6%)	3 (5.7%)	0.549
Female	10 (10.3%)	13 (9.9%)	43 (16.9%)	2 (8.7%)	0.418
Male	4 (4.1%)	13 (7.9%)	11 (3.6%)	1 (6.25%)	0.798

Values are *n* (%). *P* for trend values by logistic regression models, and *P* < 0.05 was considered statistically significant. ^†^*P* < 0.01 by *post hoc* analyses, CKD stage 1 subgroup as reference; *P* < 0.01 was considered statistically significant. CKD, chronic kidney disease; the at-risk population, male participants aged ≥50 years, and menopause female.

## Data Availability

Data are available upon reasonable request.

## Abbreviations

OP: osteoporosis
CKD–MBD: chronic kidney disease–mineral and bone disorder
KDOQI: The National Kidney Foundation's Kidney Disease Outcomes Quality Initiative
DXA: dual-energy X-ray absorptiometry
ALP: alkaline phosphatase
GLM: General linear models
FGF-23: fibroblast growth factor 23
OPG: osteoprotegerin
RANKL: receptor activator of nuclear factor- κB ligand
CKD-EPI: Chronic Kidney Disease Epidemiology Collaboration
FN: femoral neck
LS: lumbar spine
BMD: bone mineral density
CKD: chronic kidney disease
NHANES: The National Health and Nutrition Examination Survey
eGFR: estimated glomerular filtration rate
